# The Fabrication of Porous Al_2_O_3_ Ceramics with Ultra-High Mechanical Strength and Oil Conductivity via Reaction Bonding and the Addition of Pore-Forming Agents

**DOI:** 10.3390/ma18153574

**Published:** 2025-07-30

**Authors:** Ye Dong, Xiaonan Yang, Hao Li, Zun Xia, Jinlong Yang

**Affiliations:** State Key Laboratory of New Ceramics and Fine Processing, School of Materials Science and Engineering, Tsinghua University, Beijing 100084, China; callmexyz92@163.com (Y.D.); yxn20@tsinghua.org.cn (X.Y.); 18116038@bjtu.edu.cn (H.L.); zmj86644621@126.com (Z.X.)

**Keywords:** porous Al_2_O_3_ ceramics, reaction bonding, pore structure, mechanical properties, oil conductivity

## Abstract

Reaction bonding (RB) using Al powder is an effective method for preparing porous ceramics with low shrinkage, high porosity, and high strength. However, it remains challenging to optimize mechanical strength and oil conductivity simultaneously for atomizer applications. Herein, aiming at addressing this issue, porous Al_2_O_3_ ceramics with ultra-high mechanical strength and oil conductivity were fabricated via the RB process using polymethyl methacrylate (PMMA) microspheres as the pore-forming agent. The pore structure was gradually optimized by regulating the additive amount, particle size, and particle gradation of PMMA microspheres. The bimodal pores, formed by Al oxidation-induced hollow structures (enhancing bonding force) and burnout of large-sized PMMA microspheres, significantly improved mechanical strength; meanwhile, three-dimensional interconnected pores derived from particle gradation increased the diversity and quantity of oil-conduction channels, boosting oil conductivity. Consequently, under an open porosity of 58.2 ± 0.1%, a high compressive strength of 7.9 ± 0.3 MPa (a 54.7% improvement) and an excellent oil conductivity of 2.1 ± 0.0 mg·s^−1^ (a 46.5% improvement) were achieved. This superior performance combination, overcoming the trade-off between strength and oil conductivity, demonstrates substantial application potential in atomizers.

## 1. Introduction

Porous ceramic atomizers, key components for converting liquids into fine aerosol droplets through heating, have been widely applied in various fields, including medical treatment, chemical engineering, and the electronic cigarette industry [1,2,3]. Comprising a porous ceramic matrix and a resistive heating element, these atomizers function by guiding stored liquid through the matrix’s pore structure to the heating element, where the liquid is vaporized into fine droplets via resistance heating. With excellent pore structures and compressive properties, these atomizers offer advantages such as superior atomization efficiency, high stability, excellent anti-leakage properties, and extended lifespan. The thick-film process, a prevalent fabrication method, involves three main steps: preparing and machining the porous ceramic, screen-printing the resistive circuit, and vacuum (or reducing-atmosphere) sintering of the resistive paste. Previous research indicates that atomizers impose stringent requirements on the porous ceramic matrix: (1) low sintering shrinkage to prevent pore collapse and specimen deformation; (2) interconnected pores with ~60% open porosity and 1–100 μm pore size for efficient liquid storage and conduction; (3) robust mechanical properties to ensure integrity during printing and assembly; (4) high-quality resist printing for stable resistance and uniform heating; and (5) optimal atomization performance, primarily dependent on pore feature and printing quality. While screen-printing technology has matured, the influence of microstructure and properties of porous ceramics on atomization efficiency remains underexplored. Specifically, mechanical properties and liquid conductivity often trade off: enhancing mechanical strength reduces pore interconnectivity and impairs liquid conductivity, while increasing open porosity to boost liquid conduction weakens structural integrity [4,5]. This dual optimization challenge bottlenecks the development of porous ceramic atomizers as neither property can be compromised in practice.

Selecting appropriate fabrication methods is crucial to achieving interconnected pore structures that meet the requirements for mechanical strength and oil conductivity of atomizers. Notably, Huang et al. [6] showed that uniform ionic channels enhance transport properties in β″-Al_2_O_3_, analogous to oil conduction in atomizer ceramics. Feng et al. [7] demonstrated that tailored Al_2_O_3_-based microstructures optimize performance, supporting the value of structure regulation here. Common techniques for preparing porous ceramics include the addition of a pore-forming agent [8,9,10], organic foam impregnation [11,12], foaming [13,14], freeze-drying [15,16], in situ synthesis [17,18], and additive manufacturing [19,20,21,22]. Our research group [21,23,24,25,26,27] previously developed a reaction bonding (RB) process using Al powder, which exploits the Kirkendall effect [28,29] during Al thermal oxidation. This mechanism enables the in situ formation of hollow structures, as Al atoms diffuse outward faster than oxygen atoms penetrate inward through the oxide layer of Al particles. The resulting expansion, hollow structures, and bonding bridges contribute to low shrinkage, high porosity, and enhanced mechanical strength, respectively. Several related routes have been established to fabricate porous ceramics with high interconnectivity. For instance, our prior work demonstrated that selective laser sintering (SLS) with coral-like Al_2_O_3_ addition achieved an open porosity of 64.1 ± 0.4% and a bending strength of 7.37 MPa [24]. Reducing the heating rate promoted Al granule precipitation, increasing the porosity to 56.9 ± 0.6% with a bending strength of 6.5 ± 0.4 MPa [25]. Acid etching of excess Al further elevated porosity to 58.4–83.0%, with a bending strength of 2.0–15.4 MPa and compressive strength of 3.7–18.1 MPa [21]. Other studies, such as Xia et al.’s combined direct foaming and gel-freezing approach, yielded Al_2_O_3_ foams with 89.45–94.45% porosity and 0.35–2.19 MPa compressive strength [26], while Li et al. reported 87.8–94.3% porosity and 0.79–1.35 MPa compressive strength via organic foam impregnation [27]. These methods confirm the feasibility of maintaining high strength at high porosity and interconnectivity through Al oxidation hollowing. However, these methods primarily focus on pore features (e.g., open porosity, pore size distribution, pore interconnectivity) and mechanical strength regulation, with limited attention to oil conductivity. Specifically, existing RB processes lack systematic tailoring of pore features to balance mechanical strength and oil conductivity, which are both essential for reliable atomizer operation.

This study proposes a novel approach integrating Al oxidation hollowing with polymethyl methacrylate (PMMA) pore-forming agents to fabricate porous Al_2_O_3_ ceramics with bimodal interconnected pores. By systematically regulating PMMA parameters (additive amount, particle size, particle gradation), this method targets the dual optimization of mechanical strength and oil conductivity, directly addressing the unmet needs of atomizer applications. The effects of PMMA on pore structures and comprehensive properties were investigated, and the underlying enhancement mechanisms were elucidated.

## 2. Experimental Procedure

### 2.1. Raw Materials

**Figure 1:**
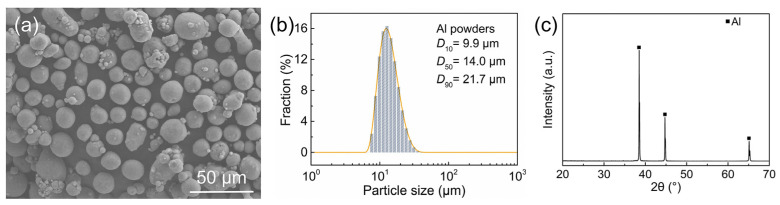
(**a**) SEM image, (**b**) particle size distribution, and (**c**) XRD pattern of Al powders.

**Figure 2:**
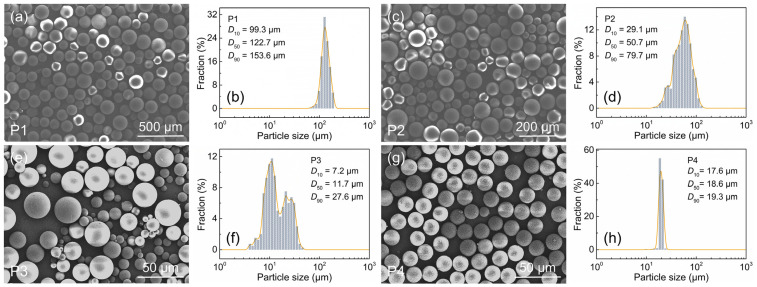
SEM image and particle size distribution of PMMA microbeads: (**a**,**b**) P1, (**c**,**d**) P2, (**e**,**f**) P3, (**g**,**h**) P4.

In this experiment, spherical Al powders (99.9%; Shanghai Yaotian New Material Technology Co., Ltd., Shanghai, China) were purchased as the raw materials, and polymethyl methacrylate (PMMA) microspheres (99.5%; Dongguan Zhangmutou Qingtian Plastic Raw Materials Business Department, Dongguan, China) as the pore-forming agents. As shown in Figure 1a–c, the spherical Al powder has a median particle size (*D*_50_) of 14.0 μm with a unimodal distribution, confirmed to be pure Al phase (JCPDS#04–0787). Four types of PMMA microspheres (labeled as P1, P2, P3, and P4) were used, with the *D*_50_ values of 122.7 μm, 50.7 μm, 11.7 μm, and 18.6 μm, respectively, as characterized in Figure 2a–h. Specifically, P1 and P4 show narrowly distributed unimodal sizes, while P2 and P3 present broadly distributed multimodal patterns. A 3 wt.% binder solution for dry pressing was prepared using polyvinyl alcohol (PVA; (C_2_H_4_O)_n_; 1750 ± 50; 99.0%; Sinopharm Group Chemical Reagent Co., Ltd., Shanghai, China).

### 2.2. Porous Ceramic Preparation

Al/PMMA composite powders were first obtained via roller ball milling (S-225, Hebei Yonglong Bangda New Materials Co., Ltd., Handan, China) at 400 r·min^−1^ for 12 h. The powders were then manually mixed with a 10 wt.% PVA solution for 10 min to ensure uniform binder distribution. The mixtures were subsequently poured into steel molds and dry-pressed at 4 MPa for 1 min to form cylindrical green samples (Φ12 mm × 12 mm) and tabular green samples (9.13 mm × 3.56 mm × 3.50 mm). After demolding, the samples were dried and strengthened at 65 °C for 6 h. Porous Al_2_O_3_ ceramics were finally obtained through a three-stage heat treatment: debinding at 600 °C for 2 h with a heating rate of 1 °C·min^−1^, pre-sintering at 1000 °C for 2 h with a heating rate of 2 °C·min^−1^, and final sintering at 1600 °C for 4 h at a heating rate of 2 °C·min^−1^.

### 2.3. Characterization

Microstructural characterization and elemental analysis were performed using a scanning electron microscope (SEM, MERLIN VP Compact, Carl Zeiss, Jena, Germany) equipped with an X-ray energy-dispersive spectroscopy detector (EDS, X-Max 50, Oxford Instruments, Oxford, UK). Particle size distributions of the powders were statistically analyzed from SEM images using Nano Measure 1.2.5 software. Phase compositions were determined by X-ray diffraction (XRD, D8 ADVANCE, Bruker, Karlsruhe, Germany) using Cu K*α* radiation (λ = 0.154 nm), with a step size of 0.02° and a scanning rate of 6°·min^−1^. The linear shrinkage (*S*) of the samples was calculated using the dimensions of the green sample (*L*_0_) and the sintered sample (*L*). The total porosity (*ε*_t_), open porosity (*ε*_o_), and relative density (*ρ*_r_) of the sintered samples were determined using the Archimedes method. Compressive strength (*σ*_c_) was evaluated on cylindrical samples via a hydraulic universal testing machine (AG-IC20KN, Shimadzu, Kyoto, Japan) at a loading speed of 0.5 mm·min^−1^, calculated using the maximum load (*F*) and the sample’s diameter (*D*). Oil conductivity (*Q*) was determined via the liquid-dropping method [30] on tabular samples and calculated using the mass of the oil droplet (*m*) and the time from initial contact to complete immersion of the oil droplet into the porous ceramic (*t*). The test oil was e-cigarette oil supplied by a collaborating enterprise, with propylene glycol and ethanol as solvents. Reported values for shrinkage, porosity, and compressive strength represent the average of four replicate samples.

## 3. Results and Discussion

### 3.1. Tunable Pore Structures and Optimized Comprehensive Properties

To meet the stringent requirements of porous ceramic atomizers, this study systematically optimized the pore structure and enhanced the comprehensive properties by fine-tuning key process parameters. Specifically, the effects of content, size, and gradation of PMMA microbeads on critical properties—including shrinkage, porosity, compressive strength, and oil conductivity—were investigated in a stepwise manner.

#### 3.1.1. Effect of PMMA Microbead Addition Amount

**Figure 3:**
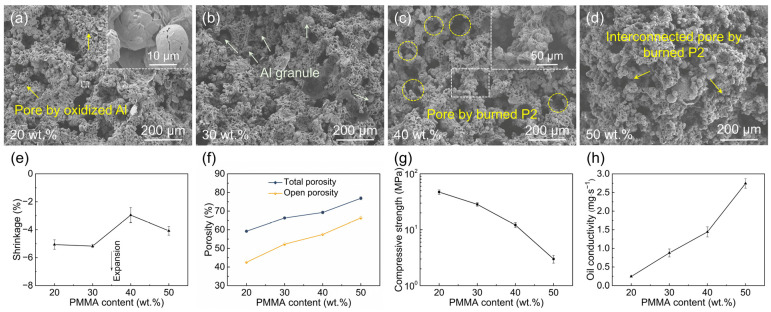
(**a**–**d**) SEM images of the fracture surface, (**e**) shrinkage, (**f**) porosity, (**g**) compressive strength, and (**h**) oil conductivity of the sintered samples with various PMMA addition amounts.

P2 microbeads with a content of 20–50 wt.% were used to investigate the effect of the PMMA microbead addition amount. As depicted in Figure 3a–d, the sintered samples contain three types of pore structures: macropores formed by the burnout of P2 microspheres, hollow spheres generated by the oxidation of Al powders, and pores resulting from particle packing. The significant disparity in particle size between Al powder and P2 microspheres leads to a bimodal pore size distribution. Most hollow spheres by Al oxidation are fractured, contributing to the open porosity of the matrix. Additionally, during Al oxidation, the diffusion of molten Al on particle surfaces and the formation of irregular bonding bridges enhance the bonding force between hollow spheres. As the P2 content increases, the volume of macropores significantly expands, with interconnected pores formed by burned P2 microspheres. Notably, Al granules larger than the original Al powders are observed within the P2-derived macropores, particularly pronounced at a 30 wt.% P2 content, which may impede pore interconnectivity and reduce the effective pore diameter.

**Figure 4:**
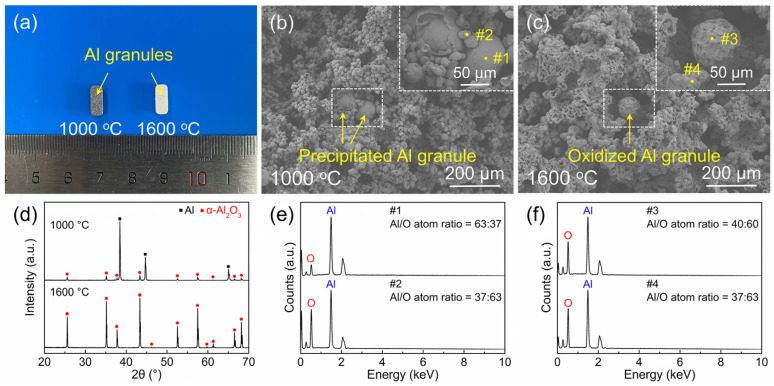
(**a**) The photograph, (**b**,**c**) SEM images of the fracture surface, (**d**) XRD patterns, and (**e**,**f**) EDS spot scanning results (Al/O atom ratios) of the samples pre-sintered at 1000 °C and sintered at 1600 °C with the P2 content of 40%.

Using a comparative analysis of samples pre-sintered at 1000 °C and sintered at 1600 °C with a 40 wt.% P2 content (Figure 4), the formation mechanism of these large Al granules is elucidated. In Figure 4a, numerous gray Al granules are observed on the surface of the pre-sintered sample, while the sintered sample shows predominantly oxidized white particles, consistent with our previous findings [25,31]. Al granule precipitation initiates around 800 °C, driven by the poor wettability between molten Al diffusing from the interior of Al particles and the surface Al_2_O_3_ layer [32]. Molten Al migrates through internal pores and aggregates on the surface under surface tension. XRD patterns (Figure 4d) reveal that at 1000 °C, the sample contains both Al and α-Al_2_O_3_ phases, with the Al phase dominating, indicating limited oxidation. At 1600 °C, the sample achieves near-complete oxidation, forming a single α-Al_2_O_3_ phase. SEM images of the pre-sintered sample fracture surface (Figure 4b) show fractured hollow spheres and large Al granules within P2-derived pores. EDS point analysis (Figure 4e) reveals Al/O atomic ratios of 37:63 for hollow spheres formed by oxidized Al powders (#2) and 63:37 for precipitated Al granules (#1). The former is close to the theoretical ratio of Al_2_O_3_ (40:60), while the latter is rich in Al. In the sintered sample (Figure 4c), more hollow spheres and denser bonding bridges are observed, and EDS analysis (Figure 4f) shows Al/O ratios approaching that of Al_2_O_3_ for both the hollow spheres (#4) and Al granules (#3). These results suggest that internal and surface Al granule precipitation share the same origin: poor wettability between molten Al and Al_2_O_3_. An increase in P2 content provides more space for Al granule precipitation, while a higher Al powder content supplies more molten Al. Consequently, the amount of precipitated Al particles initially increases but then decreases with further P2 addition.

All sintered samples exhibit negative linear shrinkage, ranging from −5.18 ± 0.10% to −2.95 ± 0.54% (Figure 3e). Referring to the conclusions of our previous research [25], dimensional changes are governed by four competing factors: (1) shrinkage from organic burnout, (2) densification-induced shrinkage during Al_2_O_3_ sintering, (3) expansion from outward Al_2_O_3_ growth, and (4) expansion due to internal stresses from Al granule precipitation. As the P2 content increases, shrinkage from organic burnout rises, while Al_2_O_3_ densification shrinkage and outward growth expansion decrease. However, the variation pattern of internal stress from Al granule precipitation with P2 content remains unclear: on one hand, the enhanced interconnectivity promotes the precipitation of Al granules; on the other hand, the reduced content of Al powders leads to a decrease in Al granule formation. These combined factors result in irregular changes in the shrinkage. Specifically, the maximum shrinkage with 40 wt.% P2 content may be related to significant shrinkage caused by organic debinding and slight expansion due to low internal stress from Al granule precipitation. Overall, the negative shrinkage effectively prevents pore collapse, preserving high porosity.

The total and open porosities increase with rising P2 content, reaching 59.2–76.9% and 42.5–66.4%, respectively (Figure 3f). The growth of P2-derived macropores and reduced pore filling by molten Al contribute to the increase in total porosity, while enhanced interconnected pores between macropores drive the rise in open porosity. An open porosity of ~60% at 40–50% P2 content aligns well with the requirements of ceramic atomizers.

The compressive strength decreases from 47.2 ± 4.2 MPa to 3.0 ± 0.5 MPa as the P2 content increases (Figure 3g), showing an inverse correlation with porosity. This decline is attributed to reduced bonding bridges between hollow spheres and weakened strut structures between macropores. According to the project requirements, the compressive strength of the porous ceramics should exceed 6 MPa. Samples with ≤40% P2 content meet the strength requirements for atomizers, whereas 50% P2 samples exhibit severe powder shedding.

The oil conductivity increases from 0.25 ± 0.02 to 2.74 ± 0.13 mg·s^−1^ with rising P2 content (Figure 3h), primarily due to enhanced open and interconnected pores. Given our experimental criteria, an oil conductivity > 1.30 mg·s^−1^ is necessary for optimal atomization. Samples with ≥40% P2 content fulfill this requirement, underscoring the critical role of pore structure in atomization performance. Considering both compressive strength and oil conductivity comprehensively, a P2 content of 40% is optimal.

#### 3.1.2. Effect of PMMA Microbead Particle Size

**Figure 5:**
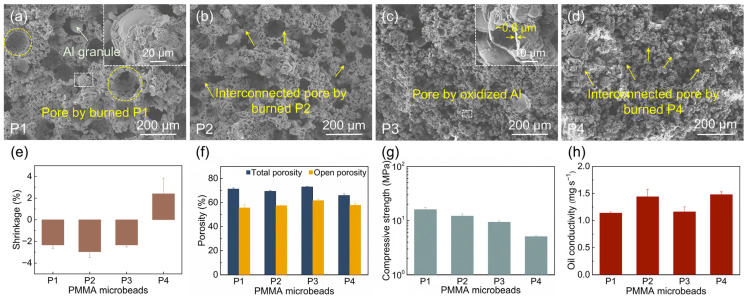
(**a**–**d**) SEM images of the fracture surface, (**e**) shrinkage, (**f**) porosity, (**g**) compressive strength, and (**h**) oil conductivity of the sintered samples with various PMMA particle sizes.

The effect of PMMA microbead particle size was investigated using four different PMMA microbeads (P1, P2, P3, and P4) with a content of 40 wt.%. As shown in Figure 5a–d, the sintered samples still consist of three types of pores. Notably, as the PMMA microsphere size decreases, the pores formed by the burnout of PMMA microspheres become smaller. No obvious changes are observed in the other two types of pores: namely, the hollow spheres formed by the oxidation of Al powder and the packing pores of Al particles. From Figure 5c, the thickness of the hollow spheres formed by Al powder oxidation is approximately 0.8 μm. The reduction in PMMA particle size leads to a looser arrangement of Al particles, weakening the bonding force between hollow spheres. Meanwhile, this also increases interconnected pores derived from the burnout of PMMA microspheres. Additionally, the precipitation of Al granules within the porous ceramics decreases, which can be ascribed to three reasons: (1) the loose arrangement of Al particles hinders the aggregation of molten Al into large granules; (2) the reduction in pore size from PMMA microsphere burnout restricts the growth of Al granules; (3) the increase in interconnected pores provides more channels for the precipitation of molten Al on the sample surface.

As shown in Figure 5e, the shrinkage of the sintered samples ranges from −2.95 ± 0.54% to 2.41 ± 1.44%. When using PMMA microspheres P1, P2, and P3, the sintered samples exhibit expansion, whereas P4-induced samples show shrinkage. The P4 microspheres feature smaller particle sizes with a narrow distribution, similar to that of Al powder, leading to a looser arrangement of Al powder in the green samples. This results in significant shrinkage during debinding, eventually causing overall shrinkage of the sintered samples. Additionally, non-uniform shrinkage leads to slight deformation of the sintered samples.

As depicted in Figure 5f, the total porosity and open porosity of the sintered samples exhibit minor variations with PMMA size, ranging from 65.9 ± 1.2% to 72.9 ± 0.4% and from 55.6 ± 2.6% to 61.7 ± 1.2%, respectively. Notably, the smallest total porosity is observed in the P4 sample, which correlates with pore structure collapse induced by substantial shrinkage. Conversely, the P1 sample exhibits the lowest open porosity due to blocked interconnected and open pores by molten Al. Overall, similar to the relevant reports [2,30], the open porosity of all sintered samples remains around 60%, meeting the requirements for ceramic atomizers.

As illustrated in Figure 5g, the compressive strength of the samples exhibits a downward trend from P1 to P4, ranging between 5.1 ± 0.2 and 16.0 ± 1.6 MPa. When the particle size of PMMA microspheres is significantly larger than that of Al powder, the sintered samples display a bimodal pore structure, featuring abundant bonding bridges between hollow spheres. The inter-pore structures formed by PMMA burnout show stronger cohesive forces, thus contributing to higher strength. The strength of all sintered samples meets the requirements for atomizers.

As shown in Figure 5h, the oil conductivity ranges from 1.14 ± 0.02 to 1.48 ± 0.05 mg·s^−1^. Particularly, the P2 and P4 samples exhibit higher oil conductivity, meeting the requirements for atomizers. The P1 samples show a lower oil conductivity due to reduced open porosity and fewer interconnected pores, while the decreased oil conductivity in P3 is attributed to the pore size and distribution of interconnected pores. The P2 and P4 samples exhibit comparable oil conductivity with similar open porosity. Specifically, while the P2 samples possess larger pores, their interconnectivity is relatively limited. In contrast, despite the smaller pore size of the P4 samples, they feature a greater number of oil-conducting channels and significantly enhanced interconnectivity. During oil infiltration in porous ceramics, both capillary force and gravity play roles, but they impose different requirements on the pore diameter of interconnected pores. Capillary-driven flow favors smaller pores for a higher driving force, while gravity-driven flow requires larger pores to reduce resistance. Therefore, optimizing both porosity and pore size distribution is critical for enhancing oil conductivity.

#### 3.1.3. Effect of PMMA Microbead Particle Gradation

**Figure 6:**
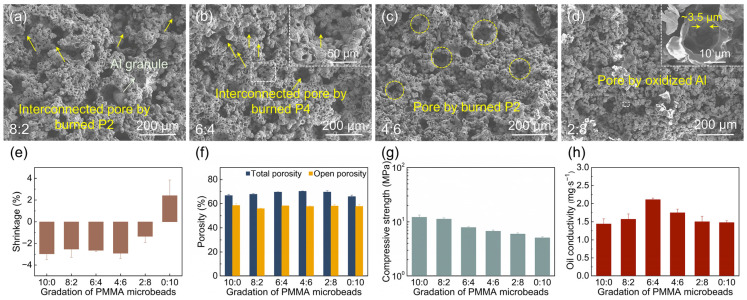
(**a**–**d**) SEM images of the fracture surface, (**e**) shrinkage, (**f**) porosity, (**g**) compressive strength, and (**h**) oil conductivity of the sintered samples with various PMMA particle gradations.

The influence of PMMA particle gradation is investigated using a content of 40 wt.% PMMA with different ratios of P2 to P4. As depicted in Figure 6a–d, as the ratio of P2:P4 decreases, the macropores formed by the burnout of P2 decrease, while the micropores generated by the burnout of P4 increase. However, the hollow spheres formed by Al powder oxidation and the packing pores among Al particles show no significant changes. According to Figure 6d, the grain size of the fractured hollow spheres formed by Al powder oxidation is approximately 3.5 μm. Compared with the single P2 microspheres, the combination of P2 and P4 microspheres enhances the interconnectivity of the macropores formed by P2 burnout, primarily due to the micropores generated by P4 burnout (Figure 6b). With the increase in interconnected pores, the number of Al granules within the porous matrix decreases significantly. Nevertheless, this also leads to a reduction in the bonding force between hollow spheres, causing the pore size distribution to gradually transform from a bimodal to a unimodal pattern.

As illustrated in Figure 6e, the shrinkage of the sintered samples ranges from −2.96 ± 0.54% to 2.41 ± 1.44%. When the P2:P4 ratio varies between 10:0 and 4:6, the shrinkage exhibits minimal change. Conversely, as the ratio shifts from 4:6 to 0:10, a significant increase in shrinkage is observed. The primary factor contributing to this substantial increase in shrinkage is the enhanced debinding shrinkage induced by the rising proportion of P4. Notably, when the P2:P4 ratio ranges from 10:0 to 2:8, the sintered samples show negative shrinkage, indicating no deformation occurred during the sintering process.

As shown in Figure 6f, the total porosity and open porosity of the samples exhibit minimal variation with the P2:P4 ratio, ranging from 65.9–70.2% and 56.0–58.2%, respectively. The open porosity of all sintered samples approaches 60%, which meets the requirements for ceramic nebulizers. This consistent open porosity indicates that adjusting the P2:P4 ratio has a limited impact on overall pore accessibility.

As illustrated in Figure 6g, the compressive strength of the sintered samples decreases with the variation of the P2:P4 ratio, spanning from 5.1 ± 0.2 to 12.1 ± 1.1 MPa. The decline in compressive strength can be attributed to two primary factors: weak bonding between hollow spheres and transition from a bimodal to a unimodal pore structure.

As depicted in Figure 6h, the oil conductivity initially increases and then decreases with the P2:P4 ratio, ranging from 1.44 ± 0.14 to 2.11 ± 0.04 mg·s^−1^. The maximum oil conductivity is achieved with the P2:P4 ratio of 6:4. This enhancement can be attributed to the optimized P2:P4 ratio, which diversifies the types of interconnected pores in the porous ceramics: pores formed by P2 and P4 burnout. The increased number of interconnected pores provides more oil-conduction channels, thereby boosting the oil conductivity. These results highlight the significant improvement in comprehensive properties enabled by particle gradation optimization. At a P2:P4 ratio of 6:4, the samples demonstrate an excellent combination of properties: a low shrinkage of −2.63 ± 0.09%, a suitable open porosity of 58.2 ± 0.1%, a high compressive strength of 7.9 ± 0.3 MPa, and a remarkable oil conductivity of 2.11 ± 0.04 mg·s^−1^. Compared with samples using only P4 microspheres, the compressive strength and oil conductivity are improved by 54.7% and 46.5%, respectively.

### 3.2. Enhancement Mechanisms of Mechanical Strength and Oil Conductivity

Optimizing the PMMA microbead addition amount, particle size, and particle gradation not only maximizes oil-conduction efficiency but also maintains favorable mechanical stability, indicating its great potential for practical applications in ceramic atomizers. The mechanisms underlying these property enhancements are analyzed and summarized as follows.

#### 3.2.1. Enhancement Mechanisms of Mechanical Strength

**Figure 7:**
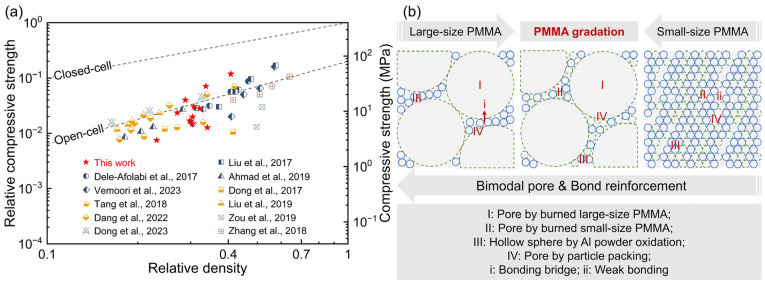
(**a**) Comparative mapping for compressive strength and relative density of open-cell Al_2_O_3_ ceramics via various processes [8,9,10,13,14,15,17,18,19,21,22]. (**b**) A schematic diagram of synergistic strengthening mechanisms in open-cell Al_2_O_3_ ceramics via reaction bonding and the addition of pore-forming agents.

Figure 7a presents a comparison of compressive strength and relative density for open-cell Al_2_O_3_ ceramics prepared via different routes. According to the Gibson–Ashby model [33], the relationship between the compressive strength (*σ_c_*) and relative density (*ρ_r_*) of porous ceramics is described by the equation:

(1) $$\sigma_{c}/\sigma_{\mathrm{fs}}\;=\;C{\rho_{r}}^{a}$$

where *σ_fs_* is the fracture modulus of dense ceramics, *σ_c_*/*σ_fs_* is the relative compressive strength, *C* is a constant, and *a* is the power-law exponent. For closed-cell ceramics, *C* and a are 1 and 1, respectively; for open-cell ceramics, *C* = 0.2 and *a* = 1.5. The porous ceramics prepared in this work exhibit compressive strengths fluctuating near the theoretical values for open-cell ceramics: slightly lower than the theoretical value at 40% P2 content, significantly lower at 40% P4 content, and with a minor decrease at a P2:P4 ratio of 6:4. Compared with open-cell ceramics prepared by various other processes (including traditional methods [8,9,10,13,14,15,17,18] and additive manufacturing processes [19,21,22]), the compressive strengths in this work are comparable or higher.

As summarized in Figure 7b, the excellent mechanical properties of the porous ceramics originate from the synergistic enhancement of bimodal pore and bonding reinforcement. The sintered samples feature four types of pore structures: pore by burned large-sized PMMA (labeled I), pore by burned small-sized PMMA (labeled II), hollow sphere by Al powder oxidation (labeled III), and pore by particle packing (labeled IV). Samples with large-sized PMMA feature pore structures I, III, and IV. Samples with P2/P4 gradation encompass structures I, II, III, and IV. Samples with small-sized PMMA contain structures II, III, and IV. Since the particle size of small-sized PMMA is similar to that of raw Al powder and far smaller than that of large-sized PMMA, the sizes of structures II, III, and IV are comparable, while structure I is significantly larger. This creates a bimodal pore size distribution in samples with large-sized PMMA, which enhances mechanical properties by hindering crack propagation paths and gradually dispersing stress during loading [34,35]. Furthermore, compared with samples using small-sized PMMA, those with large-sized PMMA exhibit tighter Al particle packing and stronger bonding bridges between hollow spheres, further reinforcing mechanical performance.

#### 3.2.2. Oil Conductivity Enhancement Mechanisms

**Figure 8:**
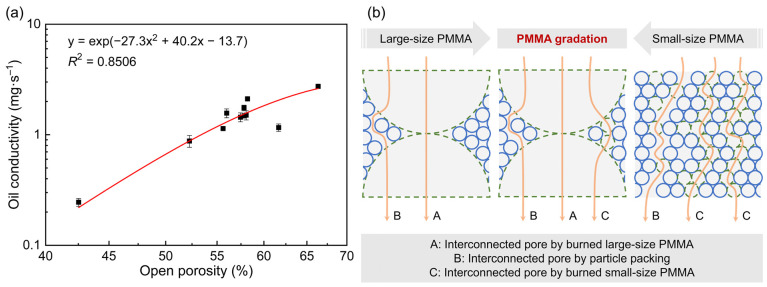
(**a**) The oil conductivity under various open porosities. (**b**) A schematic diagram showing the enhancement mechanisms of oil conductivity in open-cell Al_2_O_3_ ceramics via reaction bonding and the addition of pore-forming agents.

Figure 8a illustrates the relationship between the oil conductivity and open porosity of the porous ceramics. The experimental data are fitted using a natural exponential function, and the fitting results show good agreement with the experimental data, indicating a positive correlation between the oil conductivity and open porosity. According to the experimental results in Section 3.1, when PMMA microspheres with a single particle size are used, the oil conductivity increases with the open porosity. However, when PMMA microspheres with different particle sizes or particle gradations are applied, the correlation weakens. These findings suggest that, in addition to open porosity, other pore characteristics, such as pore size and distribution, as well as the tortuosity of interconnected pores, significantly influence the oil conductivity. The complex interplay among these factors highlights the need for a comprehensive optimization strategy to tailor the pore structures for the desired oil-conduction performance in porous ceramics.

The influence of pore characteristics on oil conductivity is clarified through model establishment and formula derivation. Under the test conditions of this work, oil infiltration along the gravitational direction aligns with the capillary force direction, meaning that the infiltration in porous ceramics is driven by the synergy of capillary and gravitational forces. Additionally, the pores of porous ceramics are simplified as a bundle of parallel cylindrical capillaries, with infiltration depth *h*, equivalent pore radius *r_e_*, effective porosity *ε_e_*, tortuosity *τ*, and cross-sectional area *A*. Assuming incompressible oil and laminar flow, Darcy’s law [36,37] describes the relationship between fluid velocity *v* and pressure difference Δ*P* as:

(2) $$v\;=\;\frac{dh}{dt}\;=\;\frac{K}{\eta }\cdot \frac{\Delta P}{L}$$

where *t* is time, *K* is the permeability (for a cylindrical capillary model, *K* = *r*_e_^2^/8), *η* is fluid viscosity, and *L* is the seepage path length (here *L* = *τh*). The pressure difference Δ*P* consists of capillary pressure Δ*P_c_* and gravitational pressure Δ*P_g_*. From the Young–Laplace equation [38]:

(3) $$\Delta P_{c}\;=\;\frac{2\sigma \cos\theta }{r_{e}}$$

where *σ* is surface tension and *θ* is the contact angle. The gravitational pressure Δ*P*_g_ is expressed as:

(4) $${\Delta P}_{g}\;=\;\rho_{l}\mathrm{gh}$$

where *ρ_l_* is the fluid density. Combining Equations (3) and (4):

(5) $$\Delta P\;={\;\Delta P}_{c}\;+\;{\Delta P}_{g}\;=\;\frac{2\sigma \cos\theta }{r_{e}}\;+\;\rho_{l}\mathrm{gh}$$

Substituting Equation (5) into Equation (2) yields the fluid velocity *v*:

(6) $$v\;=\;\frac{dh}{dt}\;=\;\frac{r_{e}\sigma \cos\theta }{4\tau \eta h}\;+\;\frac{\rho_{l}g{r_{e}}^{2}}{8\tau \eta }$$

With the initial condition *h*(0) = 0, integrating Equation (6) gives:

(7) $$h\;=\;\frac{2\sigma \cos\theta }{\rho_{l}gr_{e}}(\exp(\frac{\rho_{l}g{r_{e}}^{2}t}{8\tau \eta })-1)$$

The oil conductivity *Q* tested in this work is a mass flow rate, derived from Equation (7) as:

(8) $$Q\;=\;\frac{m}{t}\;=\;\frac{\rho_{l}\varepsilon_{e}\mathrm{Ah}}{t}\;=\;\frac{2\sigma \varepsilon_{e}\mathrm{Acos}\theta }{gr_{e}t}\;(\exp(\frac{\rho_{l}g{r_{e}}^{2}t}{8\tau \eta })-1)$$

Thus, it can be concluded that the oil conductivity *Q* is influenced by effective porosity *ε_e_*, equivalent pore radius *r_e_*, and tortuosity *τ*, with the relationship $Q\;\propto \;\frac{\varepsilon_{e}}{r_{e}}(\exp(\frac{\rho_{l}g{r_{e}}^{2}t}{8\tau \eta })-1)$. (1) The relation Q ∝ *ε_e_* indicates a linear positive correlation between *Q* and *ε_e_*. *ε_e_* directly determines the proportion of flowable pore volume in porous ceramics: a higher *ε_e_* increases the effective cross-sectional area *ε_e_A* for oil flow, proportionally enhancing the mass of oil passing through per unit time. It is important to note that *ε_e_* differs from open porosity; it represents the interconnected open porosity, as isolated pores contribute nothing to oil conduction. (2) The relation $Q\;\propto \;\frac{1}{r_{e}}\cdot (\exp(\frac{\rho_{l}\mathrm{gt}}{8\tau \eta }\cdot {r_{e}}^{2})-1)$ reveals a positive correlation between *Q* and *r*_e_: for small *r*_e_, $Q\;\propto \;\frac{\rho_{l}\mathrm{gt}}{8\tau \eta }\cdot r_{e}$, showing linear growth with *r_e_*; for large *r_e_*, $Q\;\propto \;\frac{\exp(\frac{\rho_{l}\mathrm{gt}}{8\tau \eta }\cdot {r_{e}}^{2})\;-1}{r_{e}}$, exhibiting exponential surge with *r_e_*. Here, *r_e_* represents the integrated effect of interconnected pore sizes, not the radius of isolated open pores. (3) The relation $Q\;\propto \;\exp(\frac{\rho_{l}g{r_{e}}^{2}t}{8\eta }\cdot \frac{1}{\tau })-1$ indicates an inverse correlation between *Q* and *τ*. Tortuosity *τ* (where *τ* ≥ 1, with *τ* = 1 for straight pores) characterizes pore channel bending. A larger *τ* elongates the effective flow path to *τh*, increases the frictional area between oil and pore walls, and consumes more driving force, thus limiting flow efficiency.

Based on the experimental results and theoretical analyses presented above, the enhancement mechanism of oil conductivity is illustrated in Figure 8b. The sintered samples feature three types of oil-conduction channels: interconnected pores formed by burned large-sized PMMA (labeled as A), interconnected pores from particle packing (labeled as B), and interconnected pores formed by the burned small-sized PMMA (labeled as C). Specifically, samples with large-sized PMMA contain oil-conduction channels A and B; samples with small-sized PMMA contain channels B and C; samples combining large and small PMMA microspheres contain channels A, B, and C. Channel A forms oil-conduction pathways with large pore diameters and low tortuosity, while type C channels increase the overall number of pathways. Under similar open porosity conditions, optimizing the gradation of large and small PMMA microspheres diversifies the types of pore channels. By integrating the advantages of both A- and C-type pores, a significantly higher oil conductivity can be achieved.

## 4. Conclusions

To address the critical need for balancing high mechanical strength and oil conductivity in porous Al_2_O_3_ ceramics for atomizer applications, this study developed a novel strategy integrating Al powder reaction bonding (RB) with polymethyl methacrylate (PMMA) microspheres as the pore-forming agents. The key innovation lies in the synergistic regulation of the pore structure: the RB process leverages the Kirkendall effect to form hollow structures and bonding bridges, while PMMA microspheres—with optimized addition amounts, particle sizes, and gradations—help construct a tailored pore network. This combination yields bimodal pores with reinforced bonding, enhancing mechanical strength, and three-dimensional interconnected pores with the increased number and diversity of conduction channels, boosting oil conductivity. As a result, porous ceramics achieved an open porosity of 58.2 ± 0.1%, along with a compressive strength of 7.9 ± 0.3 MPa and an oil conductivity of 2.1 ± 0.0 mg·s^−1^. Compared with the RB processes without systematic PMMA regulation (at comparable porosity), these represent improvements of 54.7% and 46.5%, respectively, resolving the trade-off between mechanical performance and oil conduction in ceramic atomizers. This work provides a viable technical route and clarifies enhancement mechanisms for fabricating high-performance porous ceramics, highlighting their substantial application potential in the atomization field.

## Data Availability

The original contributions presented in this study are included in the article. Further inquiries can be directed to the corresponding author.
